# Valuation of student-led agricultural activities at university: comparison of willingness to pay with inferred values

**DOI:** 10.1007/s11625-023-01296-2

**Published:** 2023-02-21

**Authors:** Ryo Sakurai, Takuro Uehara

**Affiliations:** College of Policy Science, Ritsumeikan University, 2-150, Iwakuracho, Ibaraki, Osaka 567-8570 Japan

**Keywords:** Willingness to pay, Inferred value, Student survey, Pro-environmental behaviors, Agriculture

## Abstract

Agricultural fields in university campuses can improve urban nutrition security, increase greenery, and provide opportunities for students to grow crops and enhance self-management skills. We conducted student surveys among freshmen in two different years (2016 and 2020) to understand their willingness to pay (WTP) for donations toward student-led agricultural activities. In order to mitigate the social desirability bias, we also asked students’ inferred WTP and compared that with conventional WTP. We found that inferred values could determine more conservative and realistic estimations of students’ donations than conventional WTP. Full model regression analysis using logit model estimation showed that students’ interest and engagement in pro-environmental behaviors increased WTP for student-led agricultural activities. In conclusion, such projects are economically feasible through student donations.

## Introduction

Creating community gardens and agricultural fields in urban areas can improve urban food and nutrition security, increase greenery in residential areas, and facilitate participatory community building to revitalize neighborhoods (Silva and Ramirez 2018; Russ and Gaus 2021). Urban agriculture can be defined as a form of farming or gardening implemented in cities, and includes community gardens, school gardens, and urban organic farms (Russ and Gaus 2021). Easy access to greenery and/or agricultural fields enhances the well-being and quality of life for urban residents (Tan et al. 2017), and provides environmental education opportunities, especially if such fields are created in the campuses of educational institutes (Pevec et al. 2017). Having greenery on campus enables students to experience nature and grow plants and crops. By independently managing such gardens and fields, they can also develop self-management and teamwork skills (Russ and Gaus 2021). Globally, having community gardens/agricultural fields on campus helps students gain knowledge about growing foods, understand food security, and obtain insights into managing and creating their own nourishment (Pevec et al. 2017; Silva and Ramirez 2018). In addition, by utilizing the recently developed concept of regenerative agriculture which aims for lower or net positive environmental and/or social impacts in terms of processes (e.g., integration of livestock) and outcomes (e.g., to increase biodiversity) (Newton et al. 2020), urban agriculture could contribute to creation of a sustainable society. However, limited research has been conducted to understand students’ perceptions about—and economic valuations of—creating agricultural fields on campus, and their willingness to get involved in such food production activities (Russ and Gaus 2021).

To respond to this gap, we conducted a survey to understand how students value efforts to create agricultural fields on campus by enquiring about their willingness to pay (WTP). We assumed that merely asking students their WTP through stated preference methods would generate biases, primarily social desirability bias, so we also asked their inferred value (i.e., how much they thought their friends would pay).

## Previous studies and theoretical background

The stated preference method continues to be one of the only valuation methods to understand the value of a service that does not necessarily have market value (e.g., starting student-led agricultural activities on campus) (Freeman III 2003; Entem et al. 2022). The most commonly used preference approach is the contingent valuation method, where individuals are asked about the level of their WTP for a certain service. Studies on valuation of ecosystem services include analysis of local residents’ WTP for river restoration (Bliem and Getzner 2012) and tourists’ WTP for environmental protection and eco-tourism (Take and Iida 2016). While the validity of contingent valuation methods is widely accepted in academia (Boyle 2003: 114), they have certain limitations. For example, these methods cannot eliminate the potential gap between hypothetical and real cash transactions. Strategies to eliminate such biases have been developed (e.g., cheap talk [making sure that respondents answer under realistic conditions] and identification of respondents’ confidence level in their answers) (Freeman III et al. 2014: 401–402). One of the most well-known limitations of general social surveys is the social desirability bias, in which respondents provide socially desirable—rather than honest—answers (e.g., overstating their contributions to charitable organizations or for protecting certain natural environments) (Champ 2003: 70; Lopez-Becerra and Alcon 2021). To mitigate such errors, the inferred valuation method was proposed (Lusk and Norwood 2009).

In inferred valuation, respondents are asked to estimate how much another person would be willing to pay in a hypothetical situation. Theoretically, it overcomes the social desirability bias, as respondents are assumed to gain little or no pleasure or satisfaction by increasing others’ social desirability (Lusk and Norwood 2009). Inferred valuation was proposed on the basis of studies that showed how people could more accurately predict their future behavior by considering other people’s behavior (rather than their own behavior) (e.g., Epley and Dunning 2000). Building on previous studies, Lusk and Norwood (2009) conducted a series of experiments and verified a significant difference between people’s predictions of their own behavior and their actual behavior. Their own behaviors were more similar to their inferred values, thus indicating that this measure provides more accurate and realistic estimates.

Research has supported inferred valuation’s ability to provide more realistic and conservative estimates than conventional WTP approaches. For example, a study in Ireland revealed that stated valuations obtained by the conventional approach were more than three times higher than inferred values; thus, it concluded that inferred valuation is promising for producing conservative estimates (Yadav et al. 2013). An investigation in Japan demonstrated that inferred valuations of the economic value of forest ecosystem services were more appropriate as they mitigated subjective valuation biases (Tanaka and Nagahiro 2019). Yet another study in Spain revealed that people stated a 2.8-fold higher WTP for protection of a coastal area than their inferred value, thereby implying significant social desirability bias (Lopez-Becerra and Alcon 2021). In Canada, people’s stated WTP for protecting at-risk species was more than two times higher than their inferred value (Entem et al. 2022).

However, while research on inferred valuation is increasing, very few studies have investigated students’ inferred values for creating sustainable campuses through participatory agricultural activities. Understanding the actual cost and how to budget for the creation of facilities such as agricultural fields enables decision-makers to evaluate the feasibility of such projects. Previous studies have shown that students were willing to donate to improve their campus environment; the estimated sum of their WTP exceeded the actual cost necessary for implementation of the project (Sakurai and Uehara 2017). If the cost of creating agricultural fields on campus, including initial and running costs, could be covered by student donations, such projects would be sustainably implemented without requiring the financial support of the university or other entities. However, supporting the creation of agricultural fields could be considered a morally and socially desirable attitude (Epley and Dunning 2000); thus, the social desirability bias means that merely asking students their WTP for such activities could produce value overestimations. Therefore, inferred valuation is necessary to explore the true value of students’ WTP in this regard.

Most, if not all, previous studies on inferred valuation implemented a one-time measurement of certain welfare. However, people’s valuation of a certain service could change depending on aspects of the context that are specific to the time when the survey was conducted. To test the validity and reliability of inferred valuation methods, we conducted studies in different years (i.e., freshmen students in 2016 and 2020) to identify inconsistencies in student valuations.

Therefore, building on previous studies that showed how inferred valuation could provide conservative and realistic estimation (e.g., Lusk and Norwood 2009; Tanaka and Nagahiro 2019; Entem et al. 2022), our first hypothesis was that students’ inferred values for donations toward student-led agricultural activities would be significantly smaller than their own subjective valuations. The second hypothesis was that inferred and subjective valuations for the same service by students in different years would be almost identical (i.e., not significantly different).

### Calculation

The theoretical background of inferred value calculation can be shown following the equation for utility [*U*] (Lusk and Norwood 2009; Tanaka and Nagahiro 2019):

1 $$U = w^{NH} M\left( {A = {\text{WTP}}^{NH} , \, H} \right) + \left( {1 - w^{NH} } \right)V\left( {I - {\text{WTP}}^{NH} , \, E} \right).$$

*M* is utility obtained by conducting socially desired behavior, and *A* indicates the actual behavior displayed. In this study, donating to student-led agricultural activities is the socially desired behavior. *H* represents the individual’s honesty level; *H* decreases as the gap between WTP and actual amount paid increases. While *V*(・) is the general utility, *I* represents income and *E* represents sustainable environmental quality for the campus in this study. If one donates, their income declines (*I* *−* WTP^*NH*^) while environmental quality increases (*E* → *E*′). *w*^*NH*^ is the weighted score that shows the relative importance of *M*(・) and *V(・)* (0 ≤ *w*^*NH*^ ≤ 1). The more the donation towards student-led agricultural activities is recognized as socially desired behavior, the bigger the value of *w*^*NH*^ affecting *M*(・). As starting student-led activities and making donations is hypothetical, *NH* (non-hypothetical) in the equation is actually *H* (hypothetical). Our analysis is based on the theoretical assumption that, as expressing willingness to donate and showing honesty both increase utility (*M*_*A*_ > 0, *M*_*H*_ > 0), hypothetical WTP will be bigger than actual WTP (WTP^*H*^ > WTP^*NH*^).

## Methods

This study was conducted among students at the College of Policy Science at Ritsumeikan University, one of the biggest private universities in Japan, with nearly 30,000 undergraduate students. Approximately 1,600 undergraduate students belong to this college, and they learn a variety of policy-science-related subjects and disciplines. Therefore, students were not necessarily studying environmental issues but had varying interests across multiple areas. The first survey was conducted on 12 April 2016 among college freshmen (*n* = 375); it was distributed in a course named “Introduction to Social Research Methods,” a mandatory course for all freshmen. Similarly, the second survey was conducted in 2020 among freshmen (*n* = 356) in the same course. We chose four years as the gap to test validity of inferred value in this study. This is because in four years, all if not most of students graduate from university; most of the students who answered the survey in 2016 as freshman were expected to graduate university by the end of 2019, and therefore, we can assume that those who answered the survey in 2020 are totally new and different groups of students. While students answered the 2016 survey in classrooms, the 2020 survey was web-based (from 31 July to 7 August 2020) as the course had shifted online because of the COVID-19 pandemic.

For both surveys, the cover letter of the questionnaire explained the contents and objectives of the research and stated that students’ participation was voluntary. Students proceeded to answer questions once they provided informed consent. The questionnaire comprised items regarding attributes (three items), interests in pro-environmental behaviors and growing organic vegetables (seven items), and actual engagement in pro-environmental behaviors (three items) (Table 1).

**Table 1:** Question items and response scale

Variable name	Description	Response scale
Interests in pro-environmental behaviors and growing organic vegetables	I am interested in recycling	1. Disagree, 2. Slightly disagree, 3. Neither agree nor disagree, 4. Slightly agree, 5. Agree
	I am interested in nature conservation on campus	
	I am interested in utilizing renewable energy	
	I am interested in using environmentally friendly goods	
	I am interested in growing my own vegetables	
	I am interested in eating vegetables I have grown	
	I am interested in eating organic vegetables	
Pro-environmental behaviors	I turn off lights as much as possible in my house	
	I bring my own bag when I go shopping	
	I set low temperatures on heaters	
Attributes	Age	1. 18, 2. 19, 3. 20, 4. 21, 5. 22 or Older
	I have a part-time job	1. No, 2. Yes
	I receive an allowance or financial support from my parents	

To determine an individual’s WTP, we used dichotomous-choice questions, which required participants to answer whether they would agree or decline to donate different amounts. Double-bound questions were used, in which participants who agreed to pay the first bid were presented with a higher bid (double the initial amount) in the following round; those who declined to pay the initial bid were shown a lower bid (half the initial amount) in the succeeding round. We distributed five versions of the survey with different bid amounts (Survey A: 500 yen [approximately $5]; Survey B: 1,000 yen [approximately $10]; Survey C: 2,000 yen [approximately $20]; Survey D: 4,000 yen [approximately $40]; Survey E: 8,000 yen [approximately $80]). The different bid amounts were created based on a previous study that investigated university students’ WTP for environmental improvements around their campus in Japan, which revealed that students’ mean and median WTP values were 2,000 and 4,699 yen (Konakayama and Sato 2004). In the survey, we first explained the hypothetical scenario (creating a student-led agricultural association) as well as actual activities that could be implemented; they were also told they could eat crops grown at the Reserve Space (Table 2). We then asked the respondents their WTP (whether they would donate the suggested amount) for being able to participate in the student-led agricultural activity. Additionally, we asked them about their inferred WTP through the question: “Would your friends and acquaintances on campus also donate?”.

**Table 2:** The scenario presented in the survey (different bid amounts are demonstrated for each survey [Survey A: 500 yen; Survey B: 1,000 yen; Survey C: 2,000 yen; Survey D: 4,000 yen; Survey E: 8,000 yen])

There is an open area called the “Reserve Space” in the southern part of the campus. We have been considering converting part of the Reserve Space into agricultural fields where students could grow vegetables for their own consumption (this is a hypothetical scenario; it is not necessarily being considered by the university). A newly created student-led agricultural association will grow crops, while students who are interested in growing crops could participate in this activity. Additionally, students who belong to the campus could eat crops grown at the Reserve Space In order to create agricultural fields (10 m * 30 m), soils, nutrition, and seeds need to be prepared. Initially, running costs for student-led agricultural activities (fees for cultivating agricultural fields and growing crops) will be paid by students who support the implementation of this initiative. However, if sufficient donations are not obtained and the student-led agricultural association is not established, the Reserve Space will continue to be an open ground If you were asked to donate XXX yen (as a one-time payment for the entire study period), would you do so? However, keep in mind that if you were to donate the requested amount, you would naturally have less money to spend on other products or services	

### Analysis

We compared significant differences between the two groups (students in 2016 and 2020) in terms of their attributes and cognitive factors by conducting chi-square and independent t-tests. We then analyzed students’ WTP and inferred values for student-led agricultural activities for the samples from 2016 and 2020 based on the simple logit model using bid as an independent variable. The logit model assumes that the difference in utility is calculated as follows: [constant (utility change created by an environmental change) + coefficient * In (bid) + error]. In addition, the rate of agreement is expressed as [1/(1 + exp(− $\Delta$*V*)] whereas $\Delta$*V* is a fixed sum of error deduced from the difference in utility. The log-likelihood function is expressed as:

2 $$\mathrm{lnL}=\sum_{i=1}^{N} \{{d}_{i}^{yy}ln{\pi }_{yy}\left(T,{T}^{U}\right)+{d}_{i}^{yn}ln{\pi }_{yn}\left(T,{T}^{U}\right)+{d}_{i}^{ny}ln{\pi }_{ny}\left(T,{T}^{L}\right)+{d}_{i}^{nn}ln{\pi }_{nn}\left(T,{T}^{L}\right)\}.$$

In this case, *d*^*yy*^ is a dummy variable, where 1 denotes respondents agreeing to donate the second bid and 0 represents all other responses. *T* is the first bid, *T*^*U*^ is the second bid when the first bid is agreed on, and *T*^*L*^ is the second bid when the first bid is declined (a lower amount). Furthermore, *N* is an observable variable. Lower and upper bounds of 95% confidence intervals were examined to identify any significant difference between these values.

Additionally, we conducted a full model regression analysis using logit model estimation to see what factors affect WTP and inferred values. Samples from 2016 and 2020 were pooled in the full model regression analysis, where all items (specifically the three attribute factors, seven factors related to interest in pro-environmental behaviors, three factors on actual engagement in pro-environmental behaviors, and years [2016 or 2020]) were used as independent variables. The functional form for the indirect utility [*V*_*ij*_] of an individual _*i*_ for alternative _*j*_ is specified in this study as:

3 $$V_{ij} = B_{0} + B_{1} AG + B_{2} PJ + B_{3} FS + B_{4} IR + B_{5} IC + B_{6} IR + B_{7} IG + B_{8} TL + B_{9} BB + B_{10} LT + B_{11} IV + B_{12} IEV + B_{13} IEOV + B_{14} YE + B_{15} log\left( {Bid} \right) + e$$

Whereas *B*_*0*_ is the intercept, *B*_*1*_ is the coefficient for age (AG), *B*_*2*_ and *B*_*3*_ are the coefficients for part time job (PJ) and financial support from parents (FS). *B*_*4*_, *B*_*5*_, *B*_*6*_, and *B*_*7*_ are the coefficients for interested in recycling (IR), nature conservation on campus (IC), utilizing renewable energy (IR), and using environmentally friendly goods (IG). *B*_*8*_, *B*_*9*_, and *B*_*10*_ are the coefficients for turning lights off as much as possible (TL), bringing my own bag (BB), and setting low temperatures on heaters (LT). *B*_*11*_, *B*_*12*_, and *B*_*13*_ are the coefficients for interested in growing my own vegetables (IV), eating vegetables I have grown (IEV), and eating organic vegetables (IEOV). *B*_*14*_ is the coefficient for year (YE), *B*_*15*_ is the coefficient for log(Bid), and *e* stands for the error.

SPSS (IBM) was used for descriptive analysis and chi-square and *t* tests. R was used to calculate WTP and inferred values and to conduct the full model regression analysis. *p* values of 0.10 were set as the significance threshold.

## Results

A majority of students in 2016 and nearly half of the students in 2020 were 18 years old; in both years, more than 90% of students were either 18 or 19 (Appendix 1). While a majority of students had part-time jobs in 2016, almost all had part-time jobs in 2020 (Table 3); chi-square tests revealed this difference was significant (*p* < 0.01). The percentage of students who received financial support from their parents was approximately 50% in both 2016 and 2020.

**Table 3:** Students who were doing part-time jobs and receiving financial support from their parents

	Frequency (%)		Chi-square score	*p* value
	2016 (*n* = 364)	2020 (*n* = 336)		
Part-time job				
Yes	271 (74.5)	303 (90.2)	29.282	< 0.01
No	93 (25.5)	33 (9.8)		
Financial support from parents				
Yes	163 (44.9)	166 (49.3)	1.330	0.249
No	200 (55.1)	171 (50.7)		

Students generally agreed that they were interested in engaging in pro-environmental behaviors, but those in 2020 showed significantly higher interests (*p* < 0.01) in recycling, conservation of campus, utilizing renewable energy, and using environmentally friendly goods (Table 4). As for behaviors, students in 2020 were more likely to answer positively to the factors “bring my own bag” and “set low temperatures on heaters” (*p* < 0.01). Meanwhile, students in 2016 were more likely to be interested in growing their own vegetables than those in 2020 (*p* < 0.05).

**Table 4:** Students’ interests and engagements in pro-environmental behaviors

		Mean	Standard deviation	*t* score	*p* value
Interests					
Interested in recycling	2020 (*n* = 336)	3.96	0.94	8.439	< 0.01
	2016 (*n* = 365)	3.34	1.03		
Interested in nature conservation on campus	2020 (*n* = 335)	3.53	1.12	3.400	< 0.01
	2016 (*n* = 365)	3.25	1.08		
Interested in utilizing renewable energy	2020 (*n* = 336)	3.91	0.95	4.109	< 0.01
	2016 (*n* = 365)	3.60	1.05		
Interested in using environmentally friendly goods	2020 (*n* = 336)	4.02	0.90	6.909	< 0.01
	2016 (*n* = 365)	3.50	1.07		
Interested in growing my own vegetables	2020 (*n* = 336)	2.75	1.30	− 2.253	0.03
	2016 (*n* = 363)	2.97	1.28		
Interested in eating vegetables I have grown	2020 (*n* = 337)	2.99	1.37	− 0.934	0.35
	2016 (*n* = 364)	3.09	1.28		
Interested in eating organic vegetables	2020 (*n* = 335)	3.23	1.29	− 0.829	0.41
	2016 (*n* = 364)	3.31	1.27		
Behaviors					
Turn off lights as much as possible	2020 (*n* = 335)	4.02	1.14	1.076	0.28
	2016 (*n* = 364)	3.93	1.20		
Bring my own bag	2020 (*n* = 337)	3.98	1.29	11.616	< 0.01
	2016 (*n* = 365)	2.77	1.48		
Set low temperatures on heaters	2020 (*n* = 337)	3.52	1.27	2.722	< 0.01
	2016 (*n* = 365)	3.26	1.33		

After excluding protest responses (e.g., those who disagreed to donations as a measure), we calculated students’ WTP for donating to student-led agricultural activities. For students in 2016, the mean WTP (truncated at the maximum bid) was 1613.6 yen (approximately $16) with a median of 1101.3 yen (approximately $11; *n* = 111). Mean inferred WTP (truncated at the maximum bid) was 1056.6 yen (approximately $10) with a median of 742.4 yen (approximately $7; *n* = 58). In both years, WTP was more than 200 yen higher than inferred values for both means and medians. However, these differences were not statistically significant, as confidence intervals of WTP and inferred values overlapped.

**Fig. 1:**
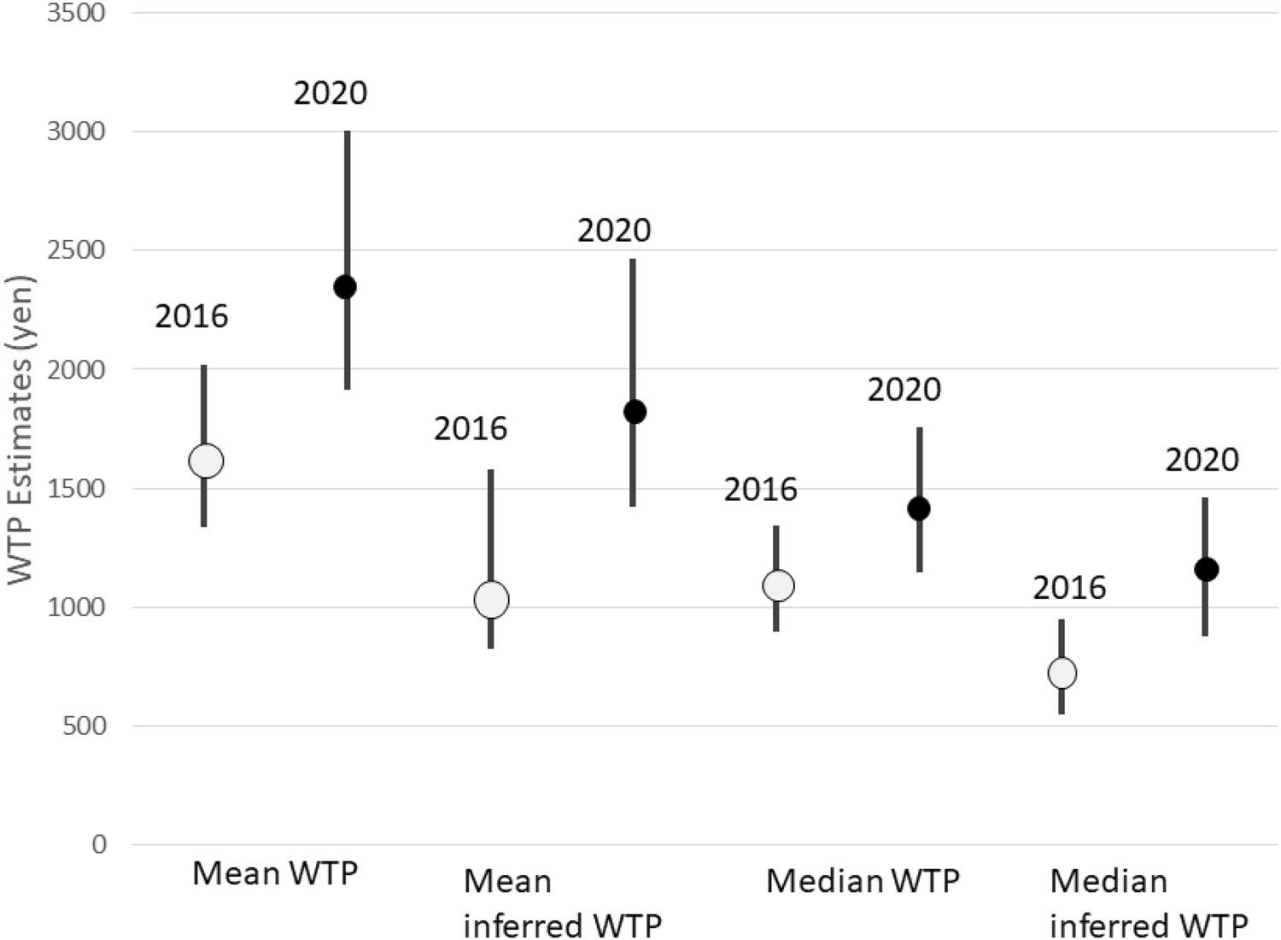
Students’ mean and median WTP and inferred values for student-led agricultural activities (CI = 95% confidence interval with lower and upper bound; *n* = 111 for WTP and *n* = 58 for inferred WTP in 2016; *n* = 107 for WTP and *n* = 66 for inferred WTP in 2020)

As for 2020, the mean WTP (truncated at the maximum bid) was 2376.8 yen (approximately $23) with a median of 1408.8 yen (approximately $14; *n* = 107). Inferred WTP (truncated at the maximum bid) was 1814.5 yen (approximately $18) with a median of 1150.6 yen (approximately $11; *n* = 66). As the confidence intervals for both the means and medians overlapped, there was no significant difference between WTP and inferred values for the year 2020 (Fig. 1).

The results of the full model regression analysis (*n* = 216) revealed that seven independent variables significantly affected students’ WTP (Table 5). Respondents who were interested in using renewable energy (*B* = 0.579, *p* < 0.01), growing their own vegetables (*B* = 0.422, *p* < 0.05), nature conservation on campus (*B* = 0.347, *p* < 0.05), brought their own bags (*B* = 0.211, *p* < 0.1), and turned lights off as much as possible (*B* = 0.050, *p* < 0.05) offered higher WTP values. On the other hand, respondents who set low temperatures on heaters offered lower WTP values (*B* = − 0.203, *p* < 0.1). In addition, independent variable of log (Bid) had significant negative influence on WTP (*B* = − 2.021, *p* < 0.01) which is understandable as respondents’ income decreases by paying higher bids which in turn decreases their utility. The mean WTP (truncated at the maximum bid) calculated from this full model analysis was 1938.97 yen (approximately $19) while the median was 1311.10 yen (approximately $13). On the contrary, the full model regression analysis for inferred values (*n* = 122) revealed that seven independent variables had a significant effect (Table 5). Respondents who received financial support from their parents gave lower inferred values (*B* = − 1.106, *p* < 05), while respondents who did part-time job (*B* = 0.892, *p* < 0.1), who were interested in using renewable energy (*B* = 0.594, *p* < 0.05), using environmentally friendly goods (*B* = 0.506, *p* < 0.1), and eating organic vegetables (*B* = 0.398, *p* < 0.1) gave higher inferred values. Respondents in 2020 offered significantly higher inferred value than those in 2016 (*B* = 0.956, *p* < 0.1). Similar to WTP, log (Bid) had significantly negative influence on inferred value (*B* = − 2.337, *p* < 0.01). The mean inferred value (truncated at the maximum bid) calculated from this full model was 1170.48 yen ($11) while the median was 857.80 yen ($8). The model that estimated WTP fit better than the one that estimated inferred value indicated by the high Log-likelihood value (− 261.806 for WTP and − 130.816 for inferred value).

**Table 5:** Factors affecting individuals’ WTP and inferred values based on the full model analysis using logit model estimation (bold letters show items that had significant effects on WTP and/or inferred value)

Variables	WTP (*n* = 216)		Inferred value (*n* = 122)	
	Estimate	*z* value	Estimate	*z* value
Intercept	10.932	8.517	10.024	5.525
Age	0.017	0.075	− 0.308	− 0.962
Part-time job	0.387	1.076	0.892*	**1.673**
Financial support from parents	0.011	0.036	− 1.106**	**− 2.504**
Interested in recycling	− 0.256	− 1.319	− 0.100	− 0.386
Interested in nature conservation on campus	0.0347**	**2.412**	− 0.028	− 1.596
Interested in utilizing renewable energy	0.579***	**3.076**	0.594**	**2.364**
Interested in using environmentally friendly goods	− 0.168	− 0.870	0.506*	**1.663**
I turn lights off as much as possible	0.050***	**2.990**	0.181	1.012
I bring my own bag	0.211*	**1.944**	0.140	0.887
I set low temperatures on heaters	− 0.203*	**− 1.731**	− 0.043	− 0.253
Interested in growing my own vegetables	0.422**	**2.138**	0.184	0.593
Interested in eating vegetables I have grown	− 0.097	− 0.456	− 0.312	− 0.950
Interested in eating organic vegetables	− 0.034	− 0.227	0.398*	**1.721**
Year	0.256	0.759	0.956*	**1.880**
Log(bid)	− 2.021***	**− 13.621**	− 2.337***	**− 10.047**
Log-likelihood	− 261.806		− 130.816	

*Significant at the 10% level, **significant at the 5% level, ***significant at the 1% level

As there were approximately 6,500 undergraduate students at the campus when this survey was conducted (nearly 1,600 at the College of Policy Science, nearly 3,500 at the College of Business Administration, and nearly 1,200 at the College of Comprehensive Psychology), the estimated utility value of implementing student-led agricultural activities, based on median WTP values calculated from the pooled 2016 and 2020 samples, is as follows:

$$\begin{aligned} & {1},{311}.{1}0{\text{ yen }} \times { 6},{5}00 \, \left( {\text{undergraduate students at the campus}} \right) \, \times \, 0.{295 }\left( {{\text{response rate }}\left[ {\text{those who showed intention to donate}} \right]} \right) \\ & \quad = { 2},{514},0{34}.{\text{25 yen }}\left( {{\text{approximately }}\$ {25},{1}00} \right). \\ \end{aligned}$$

Similarly, the estimated utility value for implementing student-led agricultural activities based on the median inferred value is as follows:

$$\begin{aligned} & {857}.{8}0{\text{ yen }} \times { 6},{5}00 \, \times \, 0.{295 }\left( {\text{same response rate as WTP}} \right) \, \\ & \quad = { 1},{644},{831}.{\text{5 yen }}\left( {{\text{approximately }}\$ {16},{4}00} \right). \\ \end{aligned}$$

## Discussion

### Comparison of students’ perceptions and demographic attributes in 2016 and 2020

By conducting a survey of students in the same college within the same grade but in different years, we were able to identify differences in their levels of environmental perceptions, degree of pro-environmental behaviors, and certain demographic attributes. Most notably, students in 2020 had significantly higher interests in engaging in pro-environmental behaviors and were more likely to engage in actual behaviors than students surveyed in 2016.

The significant differences between students’ perceptions and behaviors in 2016 and 2020 could be explained by the spread of ideas around environmental conservation and sustainability in Japanese society during these four years. For example, since 1 July 2020, Japanese stores have charged extra for plastic bags, which was a mandate based on an amendment of the Containers and Packaging Recycling Law. This encouraged consumers to bring their own bags if they wanted to avoid paying for a bag. According to a recent survey, 87% of consumers currently take their own bags when they go shopping (Planet 2021). It probably explains why students in 2020 were more likely to bring their own bags when shopping than the students surveyed in 2016.

Additionally, the Sustainable Development Goals (SDGs) were declared by the United Nations in 2015; in Japan, the cabinet is attempting to stimulate public awareness of sustainability and pro-environmental behaviors (Kanie et al. 2019). Recent media analysis has revealed that the number of newspaper articles explaining and promoting the SDGs has rapidly increased over the years, and more sustainability-related information in these media has the potential to significantly enhance citizens’ (and students’) environmental awareness and behaviors (Sakurai et al. 2021). A survey conducted among Japanese university students in 2019 revealed how the idea of SDGs has taken root; many students realized the importance of sustainability awareness and engaged in pro-environmental behaviors (Uehara and Sakurai 2021).

Moreover, the spread of COVID-19 and related lifestyle changes after the pandemic might have significantly affected people’s (and students’) perceptions on environmental issues. It has been pointed out by media and researchers that the COVID-19 outbreak is linked to the destruction of forests (Tollefson 2020). We speculate that the COVID-19 pandemic could have strengthened students’ perceived importance of environmental conservation. For example, previous studies have shown that students’ environmental awareness toward sustainability and willingness to protect the environment was enhanced after watching news about COVID-19 (Uehara and Sakurai 2021). A previous study that analyzed international survey datasets including the World Values Survey revealed how generalized trust (e.g., how much people trust others) could narrow the gap between environmental concern and pro-environmental behavior (Tam and Chan 2018), and various factors including trust level should be explored and analyzed in the future study to identify why students’ environmental concern changed over four years.

Meanwhile, significantly more students had part-time jobs in 2020 than in 2016, which could also be caused by the spread of COVID-19. Since all courses were taught online in the spring semester of 2020, students had more free time (as they did not need to commute to universities). For some courses, instructors uploaded course materials online, and students could check and study them whenever they were free without needing to attend synchronous lectures. This, in turn, gave students more flexible schedules, enabling them to engage in part-time jobs (Pustika 2020).

### Comparison of WTP and inferred values in different years

Students’ WTP for donations towards student-led agricultural activities was higher in 2020 than in 2016; both WTP and inferred values were more than 700 yen larger in 2020. Based on the fact that students in 2020 had higher interests in engaging in pro-environmental behaviors, it is reasonable that their WTP for student-led agricultural activities, which contribute to campus sustainability (e.g., by stimulating local production/consumption movements), is much higher in 2020. Several responses to open-ended questions mentioned that creating such agricultural fields on campus and starting agricultural activities is in line with the creation of a sustainable society.

The full model analysis revealed that several factors related to awareness and pro-environmental behaviors significantly affected WTP values. Our findings demonstrate how environmental awareness (e.g., energy saving) could positively affect students’ WTP for creating a sustainable campus. Meanwhile, mean WTP for creating agricultural fields in our research was 2376.8 yen, much smaller than public WTP for nature conservation calculated from previous studies in Japan [7,173 yen for conservation of fish species (Oshida and Numata 2016), 10,574 yen for use value of conservation area (Mori et al. 2013)]. While we cannot simply compare our results with previous studies as respondents’ demographics are different, it can be assumed that people’s WTP for such topic as creation of agricultural fields is generally lower than that of environmental conservation.

Contrary to our first hypothesis, the confidence intervals of WTP and inferred values overlapped for both 2016 and 2020, thus indicating that inferred values were not significantly lower than WTP. A previous study conducted among the same university students revealed that students’ WTP values were more than two times larger than inferred values for donations towards an anti-smoking patrol on campus (Sakurai and Uehara 2017); in our study, WTP was approximately 1.5 times bigger than inferred values. Lusk and Norwood (2009) reported that topics triggering stronger social desirability would result in higher WTP. Smoking is banned on campus at the university where the survey was conducted; it is also currently banned in most public areas in Japan, as the negative effects of smoking on public health are widely recognized (Inui 2007; Japanese Circulation Society 2016). Therefore, it is possible that students were more likely to state a higher WTP value compared to the inferred value for questions regarding smoking (Sakurai and Uehara 2017). Compared to the theme of smoking, which is officially banned, students would be less criticized for not supporting student-led agricultural activities. This phenomenon could have affected the results, thus yielding non-significant differences between WTP and inferred values. Previous studies on protection of nature or at-risk species all showed more than twice the difference between WTP and inferred value (Lopez-Becerra and Alcon 2021; Entem et al. 2022) implying that creation of agricultural fields is a topic that generates less social desirability bias than nature conservation. Nonetheless, even for the non-controversial topic of student-led agricultural activities, our results showed much smaller inferred values than WTP, following previous studies (Lusk and Norwood 2009; Tanaka and Nagahiro 2019). It demonstrates that inferred values could yield more conservative and potentially realistic estimates than conventional WTP for various aspects of environmental valuation.

Years did not affect students’ WTP while they affected inferred values in the full model regression analysis, implying that our second hypothesis—students’ WTP and inferred values for the same service would be identical in different years—was partially supported. This was also in line with the previous study that showed local residents’ WTP for coastal conservation projects did not significantly change over 17 years (Uehara et al. 2018), although the global value of ecosystem services more than doubled due to loss of eco-services over years (Costanza et al. 2014).

Using median values (which generally represent more conservative scores than the mean), the total utility value of creating a student-led agricultural association was more than 2.51 million yen (approximately $22,700). Approximately 300,000 yen (nearly $2,800) is necessary for preparing basic agricultural equipment (e.g., mowers, cultivators) and seeds for cultivating a small agricultural field (e.g., 10 m × 30 m). Subtracting 300,000 yen for buying necessary equipment and goods, more than 2 million yen (approximately $18,000) could be used for recruiting students to manage the agricultural fields. Based on the mean hourly part-time wage in Osaka prefecture (1,000 yen/hour; Osaka City 2021), hiring a student to work 4 h a day for 10 days a month would cost 40,000 yen. It means that 480,000 yen (nearly $4,000) is required annually to hire a student to manage the agricultural field. Therefore, our results imply that donations by students would facilitate the hiring of more than four students a year. If we use the inferred value (1.64 million yen), two students could be hired. It shows that creating on campus student-led agricultural associations and implementing agricultural activities at our study site is economically feasible via students’ donations.

As one of the first studies to reveal potential factors affecting inferred values, our goal was to reveal any factors that affect people’s valuations. However, a limitation of this study is that we were not able to delve into the reasons on why certain factors affect WTP, especially inferred value. Future research should investigate why certain factors affect inferred value, i.e., the mechanism by which people value other people’s WTP should be investigated.

## Conclusion

Our study revealed that inferred value could identify more conservative and realistic estimates of students’ donation than conventional WTP. Even using the inferred value, this study showed that student-led agricultural activities are financially feasible through student donation. Recognizing the importance of creating sustainable campuses, various efforts are made to start agricultural activities in schools and universities all over the world (Pevec et al. 2017, KASA Sustainability n.d.). Based on the results of our study, we propose policy suggestions for creating sustainable campuses. In order to realize such participatory sustainable campuses, willingness to support such projects by students and other stakeholders should be identified. When revealing their willingness to devote financially, not only WTP but their inferred value should be measured to obtain more conservative estimation avoiding social desirability bias. Once such financial value, potential donation amount, is identified, universities can let students and other stakeholders start their own organization to build such environmentally friendly campuses, and donation could be asked and collected.

## Data Availability

The anonymized data presented in this study are available on request from the corresponding author.

## Appendix 1 Grade and age of students

**Table:**

		Frequency (%)				Frequency (%)	
		2016 (*n* = 365)	2020 (*n* = 337)			2016 (*n* = 361)	2020 (*n* = 336)
Grade	First year	341 (93.4)	337 (100)	Age	18	282 (78.4)	186 (55.4)
	Second year	8 (2.2)	0 (0)		19	55 (15.1)	129 (38.4)
	Third year	9 (2.5)	0 (0)		20	12 (3.3)	17 (5.1)
	Fourth year	5 (1.4)	0 (0)		21	8 (2.2)	3 (0.9)
	Over fifth year	2 (0.5)	0 (0)		More than 22	4 (1.1)	1 (0.3)
